# Novel sustainable steel fiber reinforced preplaced aggregate concrete incorporating Portland limestone cement

**DOI:** 10.1038/s41598-024-60391-1

**Published:** 2024-05-13

**Authors:** Majed Ali Saleh, Zhihao Su, Ji Zhang

**Affiliations:** 1 School of Civil and Hydraulic Engineering, Huazhong University of Science and Technology, Wuhan, 430074 China; 2 Civil Engineering Department, College of Engineering, University of Zintan, Zintan, Libya; 3 National Center of Technology Innovation for Digital Construction, Wuhan, 430074 China

**Keywords:** Steel fiber, Preplace aggregate concrete, Two-stage concrete, Portland limestone cement, Sustainability, Civil engineering, Structural materials

## Abstract

This study proposes a novel approach by adding Portland limestone cement (PLC) to preplaced aggregate steel fiber reinforced concrete (PASFRC) to create a sustainable concrete that minimizes CO2 emissions and cement manufacturing energy usage. The method involves injected a flowable grout after premixing and preplacing steel-fibers and aggregates in the formwork. This study evaluates the mechanical properties of a novel sustainable concrete that uses PLC and steel fibers. To achieve the intended objective, long and short end-hooked steel fibers of 1%, 2%, 3%, and 6% were incorporated in PASFRC. Also, Analysis of variance (ANOVA) was used to examine the data. Results indicated that PLC and higher fiber doses increased the mechanical properties of PAC. At 90 days, PASFRC mixtures containing 6% long steel fibers demonstrated superior compressive, tensile, and flexural strengths, registering the highest values of 49.8 MPa, 7.7 MPa, and 10.9 MPa, respectively and differed by 188%, 166%, and 290%, respectively from fiberless PAC. The study confirmed the suitability and effectiveness of using PLC with steel fibers in PAC which significantly improved the mechanical properties of PASFRC. This was verified through analytical analysis and new empirical equations were proposed to predict the mechanical properties of PASFRC.

## Introduction

Preplaced aggregate concrete (PAC), often known as two-stage concrete (TSC), is a perspicuous form of concrete made by a casting procedure that differs from a standard concrete. In the context of PAC, the initial step involves the placement of the coarse aggregate within the specified moulds. Following this, as required by the specified guidelines, grout with an accepted flowability is used to fill the spaces between aggregates. The process of PAC grouting can be performed through the injection of a suitable grout, which is used as a binder, either through a pumping mechanism or by gravity. PAC contains a larger percentage of coarse aggregate, reaching around 70% of the overall concrete volume. In traditional concrete, the proportion of coarse aggregate in relation to the total volume typically ranges from 40 to 50%^1,2^. Furthermore, a lower proportion of fine particles, such as sand and cementitious materials mitigate the occurrence of creep and shrinkage, with the shrinkage being approximately 50% of conventional concrete^3^. Moreover, increased stress and direct contact between the particles of the coarse aggregate contribute to the formation of a denser matrix in PAC, resulting in higher strength^4^. Almost 70% of concrete’s load capacity is carried through coarse aggregate's point-to-point contact and then through the concrete matrix in the skeleton structure^5^. This attribute enhances the mechanical properties of PAC in comparison to ordinary concrete under similar conditions^6,7^. The pre-placement of aggregates eliminates the requirement for compaction, thereby reducing production costs. Abdelgader et al.^8^ constrained the mixture components by maintaining the volume of coarse aggregate, taking into consideration grout's workability, pumpability, and segregation. For the achievement of high-quality PAC, it is necessary to ensure thorough penetration of grout. Hence, it is imperative for grout to possess adequate fluidity in order to permeate voids without segregation.

The preplaced-aggregate technique for concrete production was developed approximately in 1937 by Lee Turzillo and Louis S. Wertz while doing restoration efforts in a Santa Fe railroad tunnel located nearby Martinez, California^9^. Since then, PAC has proven notable effectiveness across a range of construction applications, including concrete rehabilitation, mass concrete, and underwater construction^10^.

The presence of a lower cement quantity and a higher proportion of coarse aggregate in PAC results in a decrease in the demands for cement. Additionally, it has been observed that during the hydration process of PAC the heat generated during is lower than that of traditional concrete^11^. Therefore, it can be argued that PAC could be regarded as a sustainable substitute for traditional concrete. Furthermore, the growing usage of PLC in the concrete industry can be primarily attributed to its ability to enhance environmental sustainability and cost-effectiveness, decrease consumption of energy in the production of clinker, reduction of raw material consumption, and lowering carbon dioxide (CO2) emissions^12–15^ and it typically provides similar efficiency compared to Ordinary Portland Cement (OPC) throughout different applications^16,17^. PLC was initially adopted by European countries. The European Standard EN 197 allows for the inclusion of limestone as a minor additional component up to 5%. Furthermore, it categorizes PLC into four types, with types II/A-L and II/A-LL containing 6–20% limestone, and types II/B-L and II/B-LL containing 21–35% limestone^18,19^. The initial commercial production of PLCs in the United States occurred in 2005, following the performance-based specification ASTM C1157^20^. In 2007, ASTM Standard C150^21^ permitted the substitution of OPC by up to 5% limestone. In 2012, ASTM C595^22^ permitted a maximum replacement of 15% limestone for PLC. Same approach can be observed globally, such as Argentina, Brazil, Canada, China, and Mexico^23–25^.

In recent decades, PLC have seen a substantial increase in production within the cement manufacturing industry. The production of PLC involves the combining of limestone powder with OPC through blending or the process of inter-grinding cement clinker and limestone, replacing a certain percentage of the energy intensive cement clinkers with raw limestone. Several studies have shown that PLC, despite having less clinker content, can achieve comparable compressive strength as OPC^17^ and similar or enhanced durability^26^. Previous research has demonstrated that PLC has higher 28-day compressive strengths compared to OPC, as indicated by previous studies^27–29^. More extensive research of OPC and PLC with limestone contents between 6 and 16% revealed no noticeable differences in the properties of OPC and PLC^30^.

Several studies have investigated the mechanical properties and durability of PAC. However, an absence of research regarding the utilisation of PLC in preplaced aggregate steel fiber reinforced concrete (PASFRC).

The addition of steel fibers into concrete has been widely recognised to be efficient for improving ductility of concrete, minimising crack formation, decreasing crack widening, and hindering the spread of cracks through a crack bridging mechanism^31–35^. The primary advantage of incorporating fibers into concrete is the improvement of its tension performance, including post-peak strain capacity, tensile, and flexural strength^36,37^. In general, steel fiber-reinforced concrete (SFRC) mechanical properties are affected by several factors, particularly grout's properties, fiber length, shape, dose and aspect ratio^38^. Few studies have been undertaken to examine the mechanical properties of steel fiber PAC^31,39–41^. In their study, Nehdi et al.^31^ investigated the mechanical properties of PASFRC. The concrete specimens were prepared using two different sizes of steel fibers, with varying volume fractions up to 6%. The research findings exhibited significant improvements in both compressive and tensile strengths as fibers proportion increases. Furthermore, the addition of steel fiber at a dosage level of 6% led to a considerable increase in the flexural strength and flexural toughness. Multiple studies have indicated that the PAC compressive strength is predominantly affected by a number of factors, including the quality of coarse aggregates, the strength of grout, sand to binder ratio, water to binder ratio, and the presence of voids^42^.

A substantial number of studies has been conducted to study the mechanical properties of traditional concrete that includes fibers. However, there is a limited amount of research that investigated the mechanical properties of PAC produced with fibers, and as of the present time and based on the authors' extensive knowledge, there have been no research conducted on fibrous reinforced preplaced aggregate concrete that incorporates steel fibers and Portland Limestone cement in PAC. This study aims to address the lack of knowledge in this area and study for the first time the mechanical properties of preplaced aggregate steel fiber reinforced concrete (PASFRC) using for the first time Portland limestone cement PLC. Also, using various steel fiber lengths and doses, becoming the first investigation of this topic. Furthermore, new empirical equations were proposed to predict compressive, tensile and flexural strengths of PASFRC using the length and dosage of steel fibers. This provides an easy approach for designing PASFRC mixtures that include PLC and steel fibers. The results of this study will offer practitioners valuable insights to create PAFRC that are both sustainable and cost effective. This could also lead to new applications of PAC that have not been previously explored.

## Experimental program

### Materials and grout mixture proportions

**Figure 1:**
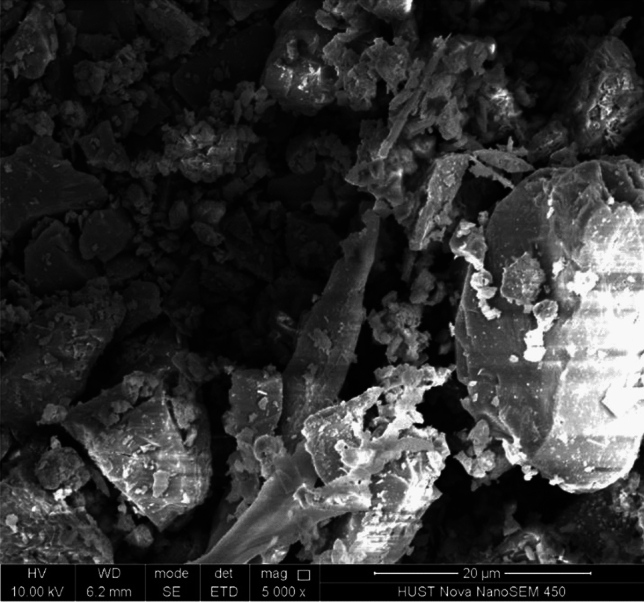
Portland limestone cement PLC under scanning electron microscope (SEM).

In the current study, Portland Limestone Cement (PLC) was utilized, which contains a specific gravity of 3.05 and a surface area of 1199 m^2^/kg. Table 1 presents the chemical composition of Portland Limestone Cement while Fig. 1 shows the microstructure of PLC under scanning electron microscope (SEM). The fine aggregate utilized in this study was silica sand, which had a fineness modulus of 2.2, a saturated surface dry specific gravity of 2.6 and a water absorption of 1.63%. Incorporating flowable grout between preplaced coarse aggregate particles makes fine sand an advantageous component in PAC. Using the absolute volume method for concrete mix design Table 2 shows the grout mixture proportions for PASFRC.

**Table 1:** Chemical analysis and physical properties of PLC.

	PLC
CaO %	59.34
SiO_2_%	20.92
MgO %	6.14
Al_2_O_3_%	4.63
SO_3_%	4.38
Fe_2_O_3_%	2.86
K_2_O %	0.77
TiO_2_%	0.28
Specific gravity	3.05
Surface area (m^2^/kg)*	1199

*1m^2^/kg = 4.882 ft^2^/lb.

**Table 2:** Grout mixture proportions.

Grout mixture NO	PLC (kg/m^3^)*	SAND (kg/m^3^)*	Water (kg/m^3^)*	HRWRA (%)
C	860	860	387	0.02

*1 kg/m^3^ = 0.06247 lb/ft^3^.

**Figure 2:**
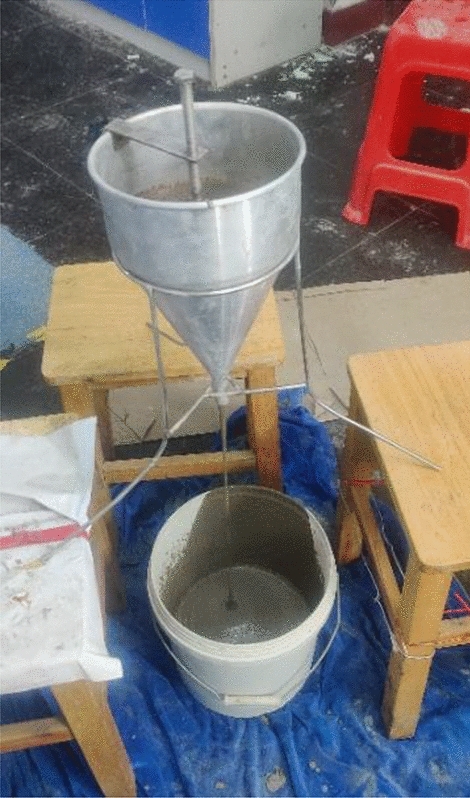
Determining the efflux time using the flow cone test.

The production of PASFRC involved the utilization of grout mixes characterized by a sand-to-binder ratio (s/b) of 1.0 and a water-to-binder ratio (w/b) of 0.45 were selected based on previous literature The grout mixture was made in accordance with ASTM C938^43^ guidelines. The measurements of mixing and flowability were carried out at room temperature (T = 23 ± 2 °C). Following the mixing process, the determination of the grout's efflux time was conducted using a flow cone test in accordance with the specifications outlined in ASTM C939^44^ (“Standard Test Method for Flow of Grout for Preplaced-Aggregate Concrete—Flow Cone Method”), which is the recognized standard test method for measuring the flow of grout for preplaced-aggregate concrete using the flow cone method. In the flow cone test (Fig. 2), the grout efflux time is measured for a grout’s volume of 1,725 mL (0.06 ft^3^) through a special cone with a 178mm diameter and a 12.7mm discharge tube.

In order to enhance the grout’s flowability, a poly-carboxylate high-range water-reducing admixture (HRWRA) with a density of 0.7 g/cm^3^ and a solid content of 96% was added into the PAC grouts at varying percentages of cement weight in accordance with the guidelines outlined in (T /CBMF 187–2022) (“Standard Specification for Solid polycarboxylates high performance water reducing admixture”)^45^. A commercialized superplasticizer called PC-303 was utilized to increase the workability by lowering the amount of mixing water required. Multiple experimental trials of grout flowability were undertaken to determine the appropriate dosage of High range water reducer admixture (HRWRA) that achieves the desired efflux time of 35–40 ± 2 s, as indicated by the (“Guide for the Use of Preplaced Aggregate Concrete for Structural and Mass Concrete Applications”)^46^. Grout mixture with HRWRA dosage of 0.02% achieved the required efflux time of 35–40 ± 2 s for successful PAC production, as indicated by the efflux time experimental results shown in Tables 3. Thus, grout mixtures with an optimal HRWRA dosage of 0.02% were chosen for additional research in the remaining portion of this study, as indicated in Table 1.

**Table 3:** Grout efflux time results.

Grout mixture efflux time (HRWRA%) (s)				Optimum HRWRA dosage (%)
HRWRA dosage (%)				
0	0.02	0.03	0.05	
45	37	31	23	0.02

**Figure 3:**
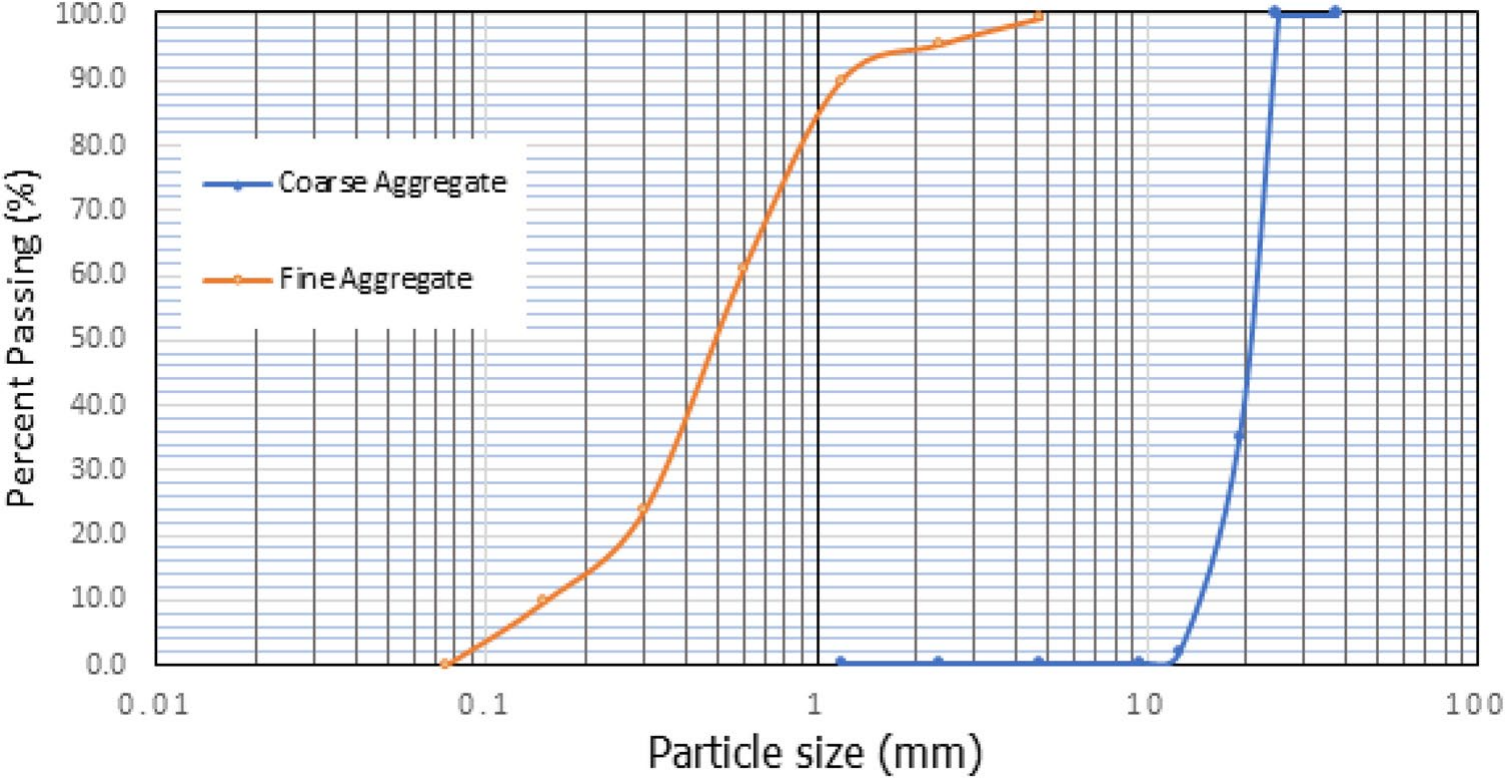
Particle size analysis of coarse and fine aggregates.

In PAC, the imposed stresses are initially transmitted to the skeleton of coarse aggregate and subsequently to the hardened grout. Thus, it is imperative to carefully analyse the right shape, size, and quality of the used coarse aggregates^10^. According to ACI 304.1^46^, it is recommended that the coarse aggregate utilized in preplaced aggregate concrete PAC undergoes a washing process to eliminate surface debris. This practice is advised in order to enhance the bonding between the aggregates and the injected grout. The coarse aggregates utilized in this study consisted of crushed granite particles, with a size distribution ranging from 9.5 to 25 mm. The coarse aggregate used in this study had a specific gravity of 2.69 and a water absorption rate of 1.07%. Figure 3 display the gradation curves for fine and coarse aggregates.

**Figure 4:**
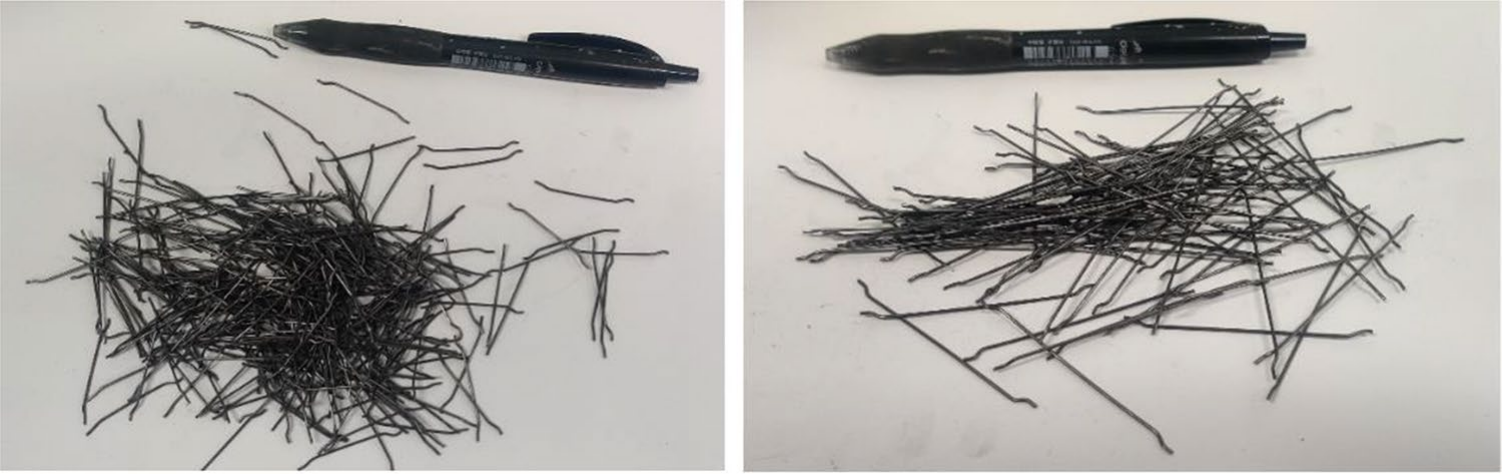
Short hooked end steel fibers (Left) and long hooked end steel fibers (right).

Two different kinds of steel fibers are used in this research. Medium monofilament hooked end steel fiber and long monofilament hooked end steel fiber with lengths of 35mm and 60 mm, 1080 MPa and 1106 MPa tensile strength, respectively and 0.75 mm diameter are utilized. Figure 4 shows a visual representation of the steel fibers (SF) while Table 4 provides their properties.

**Table 4:** Properties of monofilament hooked end steel fiber.

Fiber type	Fiber size	Length (mm)	Diameter (mm)	Aspect ratio	Tensile strength (MPa)
Monofilament hooked end	Short	35	0.75	46.67	1080
Monofilament hooked end	Long	60	0.75	80	1106

A dosage of fibers over 2.4% further reduces coarse aggregates that have been placed within the formwork^47^. However, in order to prevent the production Slurry Infiltrated Fiber Concrete (SIFCON) which doesn’t contain coarse aggregates, the present study imposes a limitation on the fiber dosage. SIFCON is a distinctive type of fiber reinforced concrete which contains a significant proportion of steel fibers, up to 20% (by volume)^48^. Therefore, the steel fiber dosage used in this study is up to 6% (by volume). The steel fiber dosages expressed as volume fractions utilized in the PAC tests were 1%, 2%, 4%, and 6%. A total of nine mixtures were formulated for the purpose of this investigation. Table 5 displays the mixture proportions of the PASFRC mixtures.

**Table 5:** Mixture proportions of the PASFRC mixtures.

Mixture NO	Steel fiber type	Steel fiber dosage (%)	S/B	W/B	HRWRA (%)
C	–	0	1.0	0.45	0.02
SL01	Long	1	1.0	0.45	0.02
SL02		2	1.0	0.45	0.02
SL04		4	1.0	0.45	0.02
SL06		6	1.0	0.45	0.02
SS01	Short	1	1.0	0.45	0.02
SS02		2	1.0	0.45	0.02
SS04		4	1.0	0.45	0.02
SS06		6	1.0	0.45	0.02

### Mixing and specimen preparation

**Figure 5:**
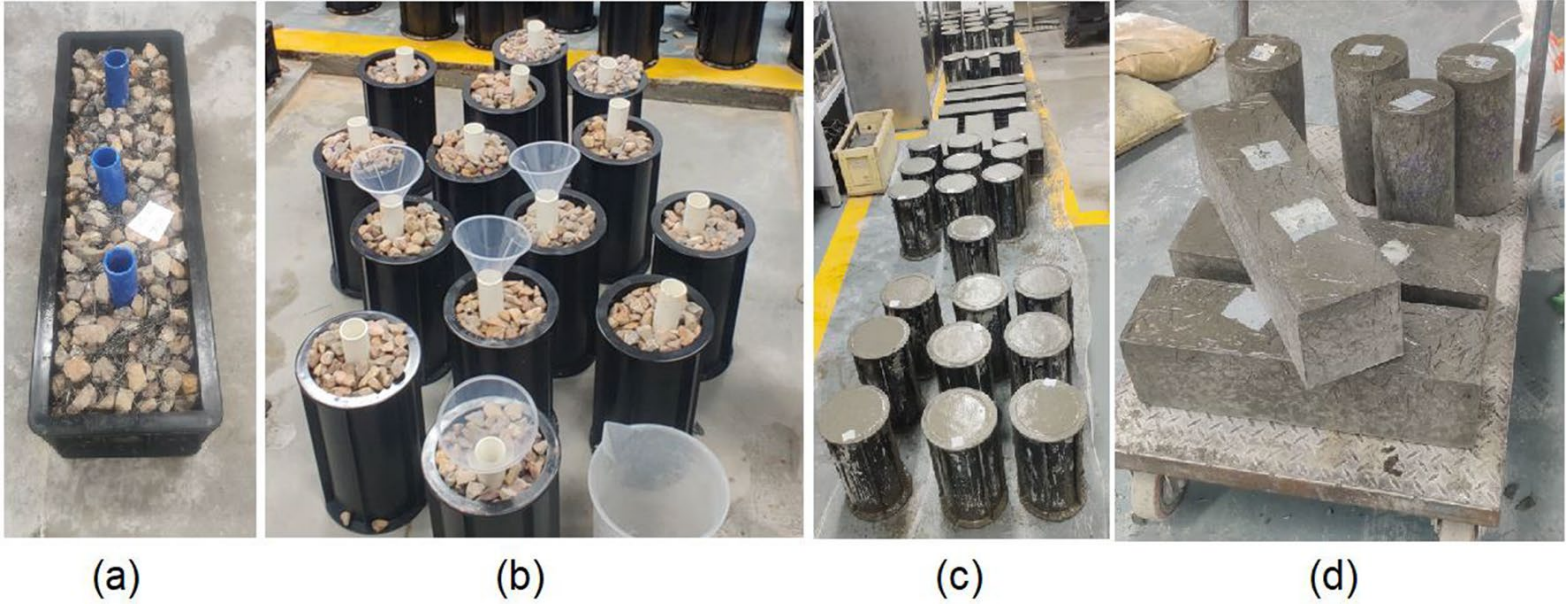
(**a**) Preplacing coarse aggregates and steel fibers with injection tubes in prism mould; (**b**) preplacing coarse aggregates and steel fibers with injection tubes in cylinder moulds; (**c**) PASFRC mixtures after grout injection; and (**d**) final demoulded specimens.

A total of 81 cylinders and 27 prisms of PASFRC were made for this research comprising of three prisms (550 mm × 150 mm × 150 mm) and nine cylindrical specimens (150 mm × 300 mm) for each mixture. Initially, the process involved the pre-mixing and pre-placement of coarse aggregates and steel fibers within the moulds while placing pipes inside to be utilized for the injection process, as shown in Fig. 5. The aggregates were not subjected to any compact loading throughout the process of grout injection.

Portland limestone cement PLC and silica sand, were subjected to a drying mixing process for a duration of 2 min using a laboratory Horizontal Single Shaft Concrete mixer. Subsequently, the addition of mixing water and HRWRA was carried out in a gradual manner to the dry mix, spanning a duration of 5 min. This process attained a state of homogeneity of the mixture. Furthermore, the cementitious grout was injected into the moulds using plastic pipes by the gravity-based method, in which the grout was poured into the moulds and the coarse aggregates and fibers then allowed the grout to infiltrate and fill all the voids. Simultaneously the moulds were placed on an automatic vibrating shaker table to get rid of all voids and to minimize the risk of honeycombing. The PASFRC samples were taken out from the moulds after a period of one day and thereafter underwent moist curing at a curing room with a temperature of 20 °C and a relative humidity of no less than 99% until reaching the age of 28 days.

### Experimental procedures

The cylindrical specimens were subjected to testing after 28 days in order to determine the compressive strength, modulus of elasticity and splitting tensile strengths of PASFRC. The compressive strength was assessed using (“Standard Practice for Making Test Cylinders and Prisms for Determining Strength and Density of Preplaced-Aggregate Concrete in the Laboratory”)^49^. Modulus of elasticity evaluated using T 0557-2005 (“Standard Test Method for Static Modulus of Elasticity of Concrete in Compression of Cylindrical Concrete Specimens”)^50^ and splitting tensile strengths of PASFRC cylinders assessed using ASTM C496 (“Standard Test Method for Splitting Tensile Strength of Cylindrical Concrete Specimens”)^51^.

For the compressive strength test the load was consistently applied at a rate of 0.25 MPa/s until the point of failure. The calculation of the compressive strength of each specimen involved dividing the maximum load applied on the specimen by its cross-sectional area.

**Figure 6:**
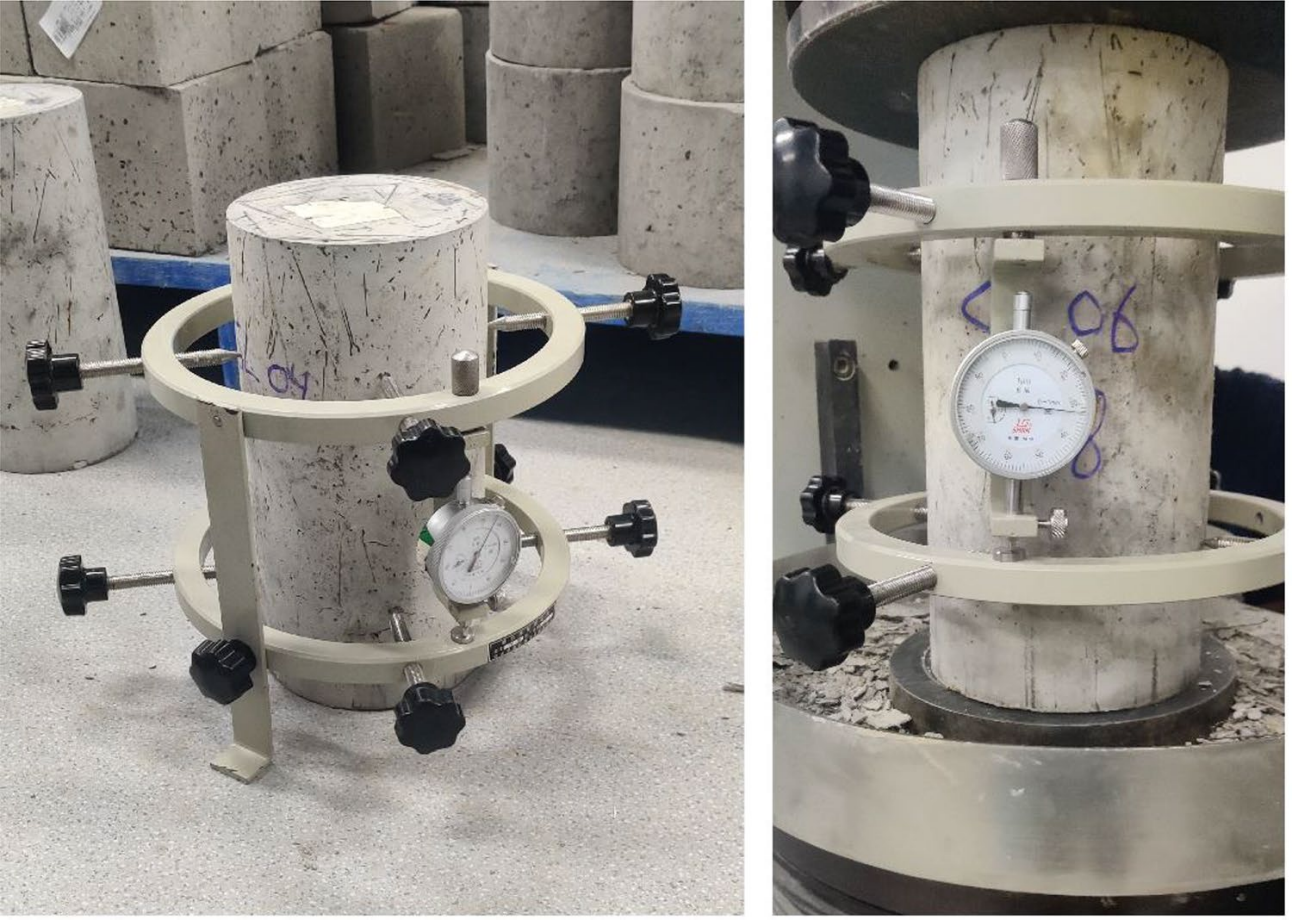
Modulus of elasticity test setup.

Modulus of elasticity test conducted in compliance with the Chinese standards for static modulus of elasticity of cylindrical concrete specimens^50^. To measure the modulus of elasticity a compressometer was fastened to every sample with two dial gauge indicators at opposite sides. Figure 6 illustrates the modulus of elasticity test setup. The following equation are used to determine the modulus of elasticity:

1 $${E}_{c}=\frac{4({F}_{a}-{F}_{0})}{{\varvec{\uppi}}{\mathbf{d}}^{2}}\times \frac{L}{\Delta n}$$

where ${E}_{c}$= concrete modulus of elasticity, (Mpa); ${F}_{a}$= final load (corresponding load value at $\frac{1}{3} Failure Load$), (N); ${F}_{0}$= initial load (corresponding load value at 0.5 Mpa), (N); L = measuring distance, (mm); d = calculated diameter of the specimen, (mm); and $\Delta n$ = At the last loading, the mean value of deformation difference on both sides of the specimen under ${F}_{a}$ and ${F}_{0}$, (mm).

2 $$\Delta n=\frac{({{{\varvec{\upvarepsilon}}}^{left}}_{a}+{{{\varvec{\upvarepsilon}}}^{{\text{right}}}}_{a})}{2}-\frac{({{{\varvec{\upvarepsilon}}}^{left}}_{0}+{{{\varvec{\upvarepsilon}}}^{{\text{right}}}}_{0})}{2}$$

where ${{\varvec{\upvarepsilon}}}_{a}$ = Deformation of specimen at ${F}_{a}$ time scale, (mm); and ${{\varvec{\upvarepsilon}}}_{0}$ = Deformation of specimen at ${F}_{0}$ time scale, (mm).

In the splitting tensile test, PASFRC specimens were horizontally positioned and the rate of loading was controlled within the specified range of 0.7 to 1.4 MPa/min. The load was steadily applied until the point of failure using indirect tension, namely through the mechanism of splitting along the vertical axis. The equation used for the determination of the tensile strength was as follows:

3 $$ft=\frac{2P}{\pi ld}$$

where $ft$ = splitting tensile strength, (MPa); *P* = maximum applied load indicated by the testing machine, (N); *l* = length of the specimen, (mm); and *d* = diameter of the specimen, (mm).

The flexural performance of PASFRC prisms was evaluated after 28 days by a three-point bending test, following the specifications outlined in ASTM C1609 (“Standard Test Method for Flexural Performance of Fiber-Reinforced Concrete-Using Beam with Third Point Loading”)^52^. The force of loading was applied in a direction perpendicular to the direction of the grouting procedure. The ultimate load or peak refers to the maximum load recorded on the load-mid span displacement curve. The modulus of rupture was determined following the guidelines provided in ASTM C1609.

The determination of the modulus of rupture was conducted utilizing the subsequent formula:

4 $$R=\frac{PL}{b{d}^{2}}$$

where *R* = the modulus of rupture, MPa [psi]; *P* = the maximum load, N [lb.]; *L* = the span length, mm [in.]; *b* = the average width of the specimen at the fracture, as oriented for testing, mm [in.]; and *d* = the average depth of the specimen at the fracture, as oriented for testing, mm [in.].

Additionally, the results of experiments were subjected to analysis of variance (ANOVA) for analysis. The statistic F value is obtained in order to evaluate the statistical significance of an experimental variable, for instance steel fiber dose or fiber length. This value reflects the ratio between the mean squared error obtained from different variances, such as different lengths of steel fiber, to the mean squared error recorded within treatments. The incorporation of multiple samples in the analysis, as opposed to depending on one single sample, serves to address the inherent variability present within treatments. The F values attained in each ANOVA test was consequently compared to a specified critical F-value obtained from statistical tables, which is established depending on the chosen significance level (α), degree of freedom of variation between treatments (a-1) and degree of freedom of error (within treatments) (N-a).

The significance level is a measure of statistical significance that measures the probability of rejecting the null hypothesis when it is actually true. Moreover, the determination of the critical F value is dependent upon the degrees of freedom of error between treatments, which are derived from the number of treatments and observation present in the experiment. In this research a significance level of 0.05 is chosen, as it is commonly employed in civil engineering research due to its established reliability. When the value derived from the probability density function of an F-distribution surpasses the critical value, it signifies that the variable under consideration has a statistically significant influence on the mean of the outcomes^53^.

## Results and discussion

### Compressive strength

**Figure 7:**
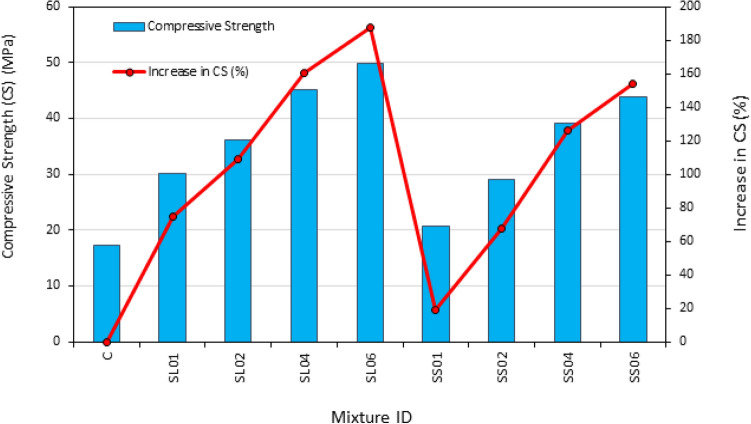
Compressive strength results of PASFRC.

Table 6 and Fig. 7 illustrates the range of compressive strength values after 28 days obtained for the 9 PASFRC mixtures, with values ranging from 17 to 50 MPa. In general, the addition of steel fibers led to a substantial increase in the compressive strength of PAC specimens. The data indicates a positive correlation between fiber dosage increase and PASFRC compressive strength. Based on the data presented in Fig. 7, the control specimen denoted as "C" exhibited a compressive strength of 17.3 MPa. In comparison to the control mixture without steel fiber dosage C, the mixtures SL01 and SL02, which contained 1% and 2% of long end-hooked steel fibers, both exhibited a substantial increase in compressive strength of 74.6% and 109.2%, respectively. Meanwhile, the addition of short hooked-end steel fibers in the PASFRC specimens (namely SS01 and SS02) at dosages of 1% and 2%, resulted in a respective increase of compressive strength by 19.1% and 67.6%, respectively when compared to the reference specimens C. The SL06 mixture had the highest compressive strength, reaching a significant strength of 49.8 MPa. This is a substantial increase of 187.9% in comparison to mixture C. The high surface area of PLC contributed in increasing the compressive strength of PAC compared to previous research studies that used OPC^31,54,55^.

**Table 6:** mechanical properties of PASFRC mixtures after 28 days.

PASFRC mixture ID	Compressive strength		Split tensile strength		Flexural strength		Modulus of elasticity	
	(MPa)*	COV %	(MPa)*	COV %	(MPa)*	COV %	(GPa)*	COV %
C	17.3	2.86	2.9	3.32	4.4	11.01	22.1	16.6
SL01	30.2	1.93	3.6	3.67	4.7	5.98	35.2	4.6
SL02	36.2	3.87	4.3	2.88	7.2	7.57	10.6	3.8
SL04	45.1	3.69	6.3	9.14	10.9	7.74	9.4	7.5
SL06	49.8	0.83	7.7	4.21	16.0	4.53	6.7	4.4
SS01	20.6	2.33	2.3	3.30	4.2	1.51	14.3	15.8
SS02	29.0	1.87	3.5	8.69	4.4	4.32	10.5	6.7
SS04	39.1	1.73	3.7	10.09	5.9	6.03	6.8	13.6
SS06	43.9	6.04	4.2	0.56	7.4	9.09	9.0	30.1

*1 MPa = 0.001; GPa = 0.145038 ksi.

Typically, the steel fiber dosage in traditional steel-fiber reinforced concrete (SFRC) is restricted to 2% as a result of deficient workability and the need to provide a uniform dispersion of steel fibers^47,56^. Therefore, increased fiber doses, beyond 2%, have a tendency to result in the formation of clusters and fiber balls. This, consequently, leads to the occurrence of voids and "honeycombing" in the mixture, which ultimately leads to a reduction in its strength and a greater vulnerability to microcracks^57^. On the contrary, the PASFRC mixing technique effectively solves these issues by first placement of fibers with the coarse aggregates in the form prior to the grout injection procedure. Therefore, by excluding the Slurry Infiltrated Fiber Concrete (SIFCON) mixing method, which does not incorporate coarse particles, it is possible to achieve a greater fiber dosage of 6% without encountering the aforementioned issues. Hence, this leads to the successful achievement of greater compressive strength results in PASFRC.

**Figure 8:**
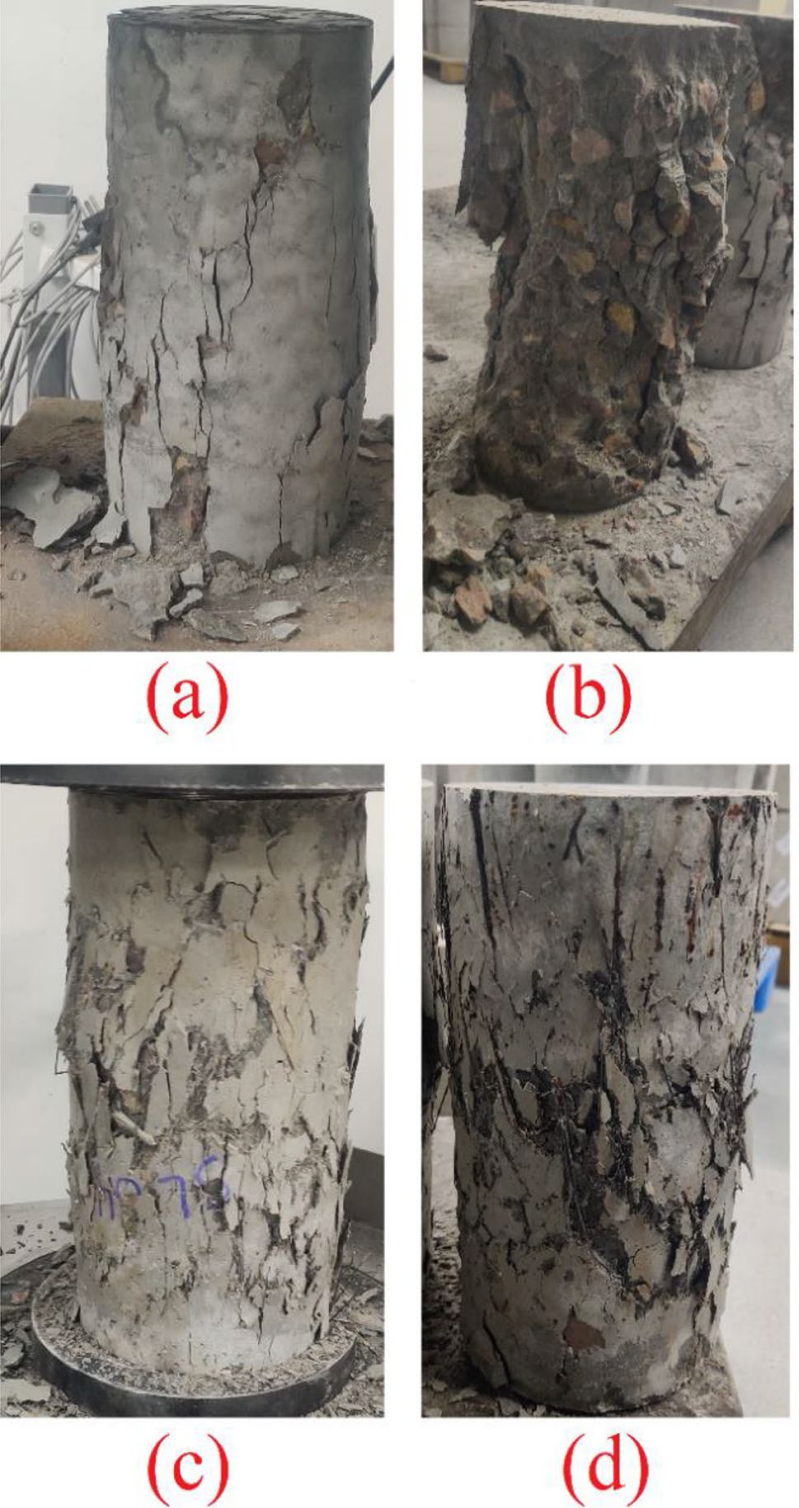
Failure modes of the tested specimens (**a**) control mixture, (**b**) control mixture, (**c**) SL04, (**d**) SL06.

Figure 8 illustrates the failure patterns of compression-tested cylinders with and without fibers. In the control samples (Fig. 8a,b), splitting cracks along the height and localized crushing at the top/bottom ends are evident. Crushing and splitting predominantly occur in all control specimens, similar findings have been documented in prior research^58–60^. Additionally, fibrous PAC concrete mixtures exhibited significantly less spalling than the non-fibrous control mixture.

The addition of hooked end steel fiber to the concrete altered the failure mode to shear failure (Fig. 8c,d). This is attributed to the enhanced bonding and tensile resistance provided by the fibers, altering the progression of vertical cracks into inclined shear cracks. Increasing the fiber dosage from 2 to 6% mitigated the severity of shear cracks and promoted a more distributed crack pattern, facilitating a more uniform distribution of tensile stresses internally. Similar failure observations have been reported previously^54,61^.

The SS04 and SS06 mixtures, which contained 4% and 6% short end-hooked steel fibers respectively, exhibited large increases in compressive strength compared to the SS02 mixture, which contained 2% short steel fibers. In particular the SS04 mixture achieved a 34.8% increase in compressive strength, while the SS06 mixture gained a 51.4% increase compared to SS02. Additionally, it was observed that the SL04 and SL06 mixtures exhibited a 24.6% and 37.6% increase in compressive strength respectively, compared to SL02 mixture. The observed outcome can be ascribed to the correlation between the dosage of steel fibers and the improvement of crack resistance and propagation, resulting in an increase in compressive strength^56,62,63^. This is confirmed by the analysis of variance (ANOVA) conducted at a significance threshold of α = 0.05, as shown in Table 7, provided validation that the change in fiber dose had a significant impact on the compressive strength results. The obtained F value of 94.88 for the overall compressive strength data was found to be significantly greater than the essential F value of 6.61 $({F}_{\mathrm{0.05,1},5})$ and the P-value was 0.0002 which is lesser than 0.05. P-values below the threshold of 0.05 indicate that the model terms are statistically significant. As stated by Montgomery^53^, when the critical value of the F-distribution density function is exceeded, it signifies that the evaluated variables have significant effects on the results. Based on ANOVA’s analysis the absolute fraction of variance (R^2^) was 0.957. Therefore, below is a proposed empirical formula based on the length and dosage of steel fibers for predicting the compressive strength of PASFRC:

5 $${\varvec{f}}{{\varvec{c}}}^{\mathrm{^{\prime}}}= 9.33106 + 0.287\mathrm{ l }+4.23814{f}_{d}$$

where ${\varvec{f}}{{\varvec{c}}}^{\mathrm{^{\prime}}}$= compressive strength of PASFRC (MPa); ${\text{l}}$= steel fiber length (i.e., 35 or 60 mm); and ${f}_{d}$= Steel fiber dosage (i.e., 1,2,4, or 6%).

**Table 7:** Analysis of variance (ANOVA).

Mechanical properties of PASFRC	Effect of fiber dosage			Effect of fiber length			R^2^
	$F$	${F}_{(\mathrm{0.05,1},5)}$	p-value	$F$	${F}_{(\mathrm{0.05,1},5)}$	P-value	
Compressive strength	94.88	6.61	0.0002	18.44	6.61	0.0078	0.9627

Mechanical properties of PASFRC	Effect of fiber dosage			Effect of fiber length			R^2^
	$F$	${F}_{(\mathrm{0.05,1},4)}$	p-value	$F$	${F}_{(\mathrm{0.05,1},4)}$	P-value	
Tensile strength	86.69	7.71	0.0007	80.77	7.71	0.0008	0.9778
Flexural strength	540.5	7.71	< 0.0001	370.43	7.71	< 0.0001	0.9961

The findings of the study revealed that long end-hooked steel fibers with 60 mm in length, conveyed greater compressive strength compared to short end-hooked steel fibers measuring 35 mm in length. For instance, the PASFRC specimens, which was reinforced with a steel fiber dose of 1% (i.e., SL01 and SS01), exhibited a difference of 46.6% in compressive strength. An inverse relationship was noticed between the steel fiber length and the PASFRC's compressive strength. As the steel fiber doses increase, the variation in compressive strength between long and short fibers for a given dosage decreases. There was a notable disparity in compressive strength observed between SL02 and SS02, with SL02 exhibiting a 24.8% higher compressive strength compared to SS02. In a comparable manner SL04 displayed a 15.3% greater compressive strength than SS04. The SL06 and SS06 combinations exhibited the least disparity in compressive strength, with only 13.4% difference. These results have been verified and validated by the statistical analysis, specifically the analysis of variance (ANOVA), The computed F-value of 18.44 for the compressive strength exceeded the corresponding critical F value of 6.61 $({F}_{\mathrm{0.05,1},5})$. Which means that the variability in fiber length had a significant impact on the mean of the overall compressive strength results, as indicated in Table 7.

### Modulus of elasticity

**Figure 9:**
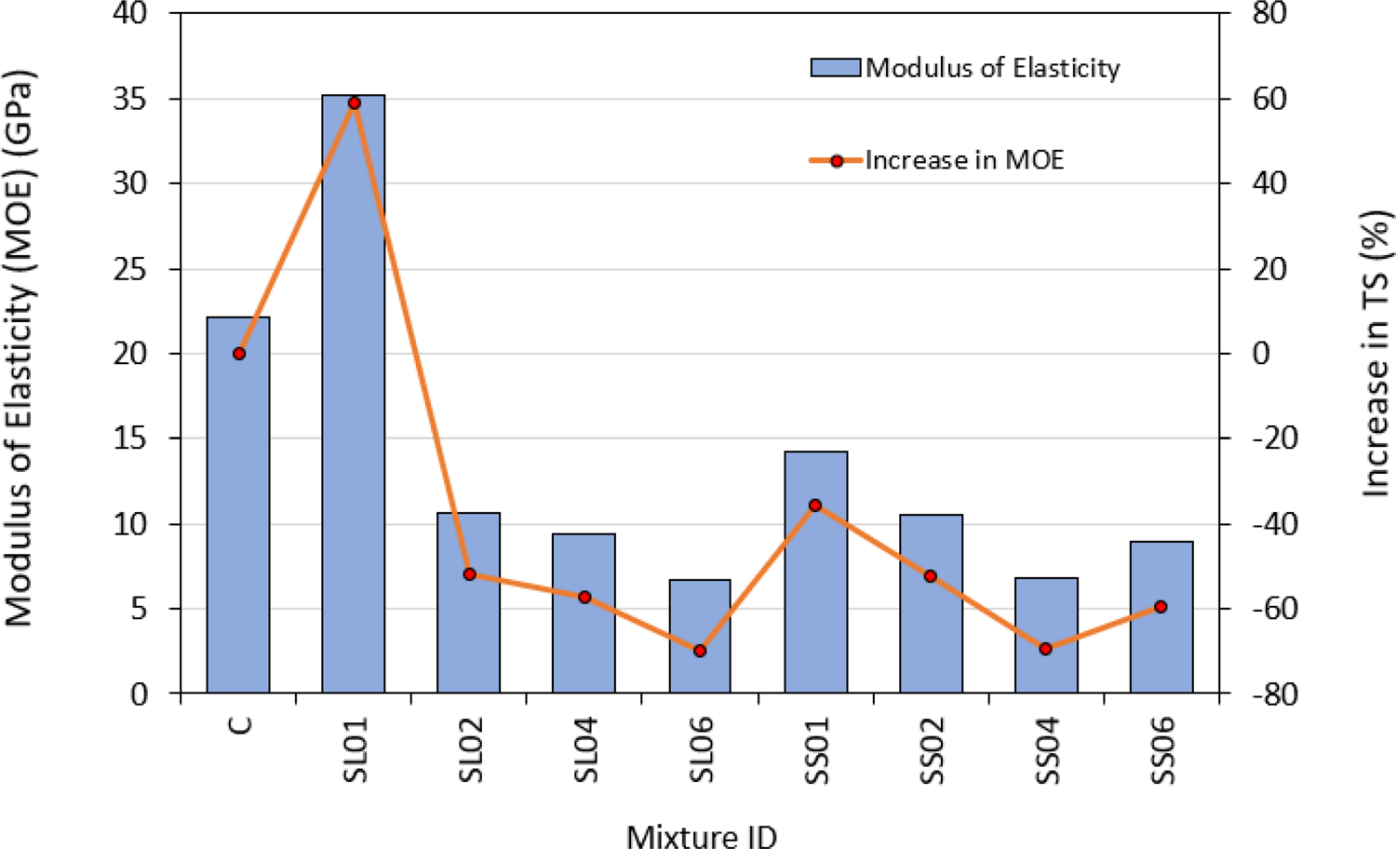
Modulus of elasticity results of PASFRC.

The modulus of elasticity (MOE) holds significant importance in the properties of concrete, since it exerts a profound influence on the performance and efficacy of concrete constructions. The MOE results of the PASFRC mixtures at 28-days are presented in Table 6 and Fig. 9. As predicted, the incorporation of steel fibers into the PASFRC mixes led to a notable decrease in the elastic modulus in comparison to the control mixture C without fibers. The elastic modulus of SS01, SL02, SS02, SL04, SS06, SS04, and SL06 mixtures exhibited a decrease in comparison to the control mixture of approximately 35.5%, 52.1%, 52.5%, 57.4%, 59.3%, 69.2%, and 69.8%, respectively.

The MOE of PASFRC demonstrated a decrease as steel fiber doses increased. The observed decrease in MOE of the PASFRC mixes is attributed to the rise in porosity resulting from the addition of fibers. This claim is corroborated by Nehdi et al.^55^ and Alfayez et al.^54^ findings, who observed a comparable reduction in the modulus of elasticity (MOE) when steel wire fibers were incorporated into PAC, in contrast to the fiberless control mixture. However, SL01 mixture exhibited a 59% increase in comparison to the control mixture. The MOE is influenced by several factors, such as the characteristics of the cement paste, the stiffness of the aggregates used^64,65^, modulus of elasticity of aggregates, steel fibers geometric size and volume fraction, and the concrete’s porosity^56,65–68^.

As stated by Najjar et al.,^10^ a positive correlation was noticed between MOE and compressive strength. In general mixes of long steel fibers had better results in comparison to short steel fibers mixtures with the same dosage. For instance, SL01, SL02, and SL04 specimens revealed an increase in modulus of elasticity of around 146%, 1%, and 38% when compared to SS01, SS02 and SS04 specimens, respectively.

### Splitting tensile strength

**Figure 10:**
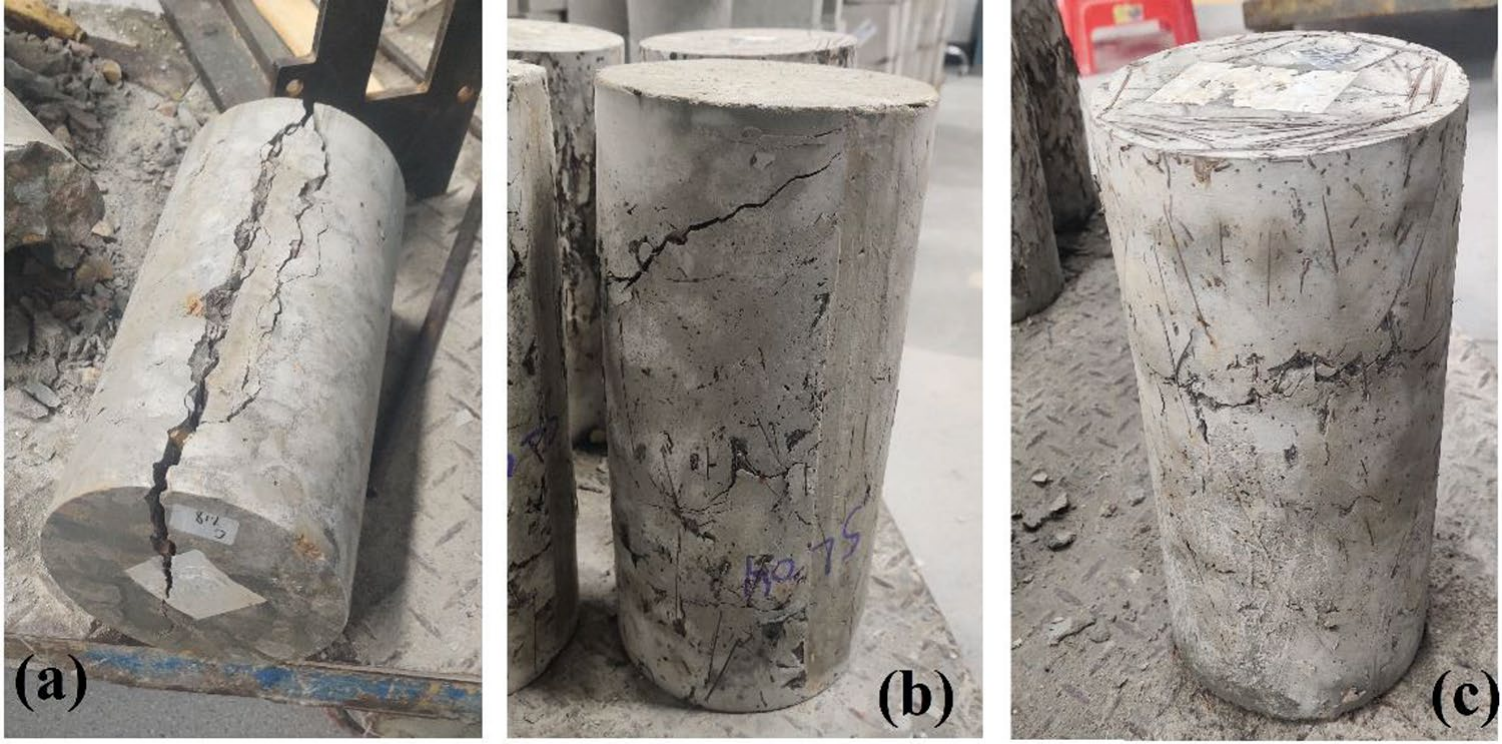
Failure mode in split tensile strength test of PASFRC specimens (**a**) control mixture, (**b**) SL04, (**c**) SL06.

Figure 10 illustrates the failure mode observed during the split tensile strength test. In non-fibrous specimens (Fig. 10a), failure occurred as the specimens split into two halves, exhibiting a large damage zone. In contrast, PASFRC specimens remained intact even after reaching peak load, as shown in Fig. 10b. The presence of fibers in PASFRC acts as a bridging element during the splitting process, transferring load from the matrix to the fibers and thus enabling them to bear additional load compared to fiberless PAC specimens, resulting in improved split tensile values.

As the fiber volume increases, concrete's failure mode transitions from shear to flexure, leading to prolonged crack formation times^54,61,69–71^. Additionally, specimens with 6% steel fibers (Fig. 10c) exhibit surface cracks localized in the centre of the concrete, with minimal through cracks. The fracture surface reveals aggregates and cement matrix interconnected by steel fibers. Corroborating findings reported by Jin et al.^72^ documented exceptionally strong bond strengths between steel fibers and concrete.

**Figure 11:**
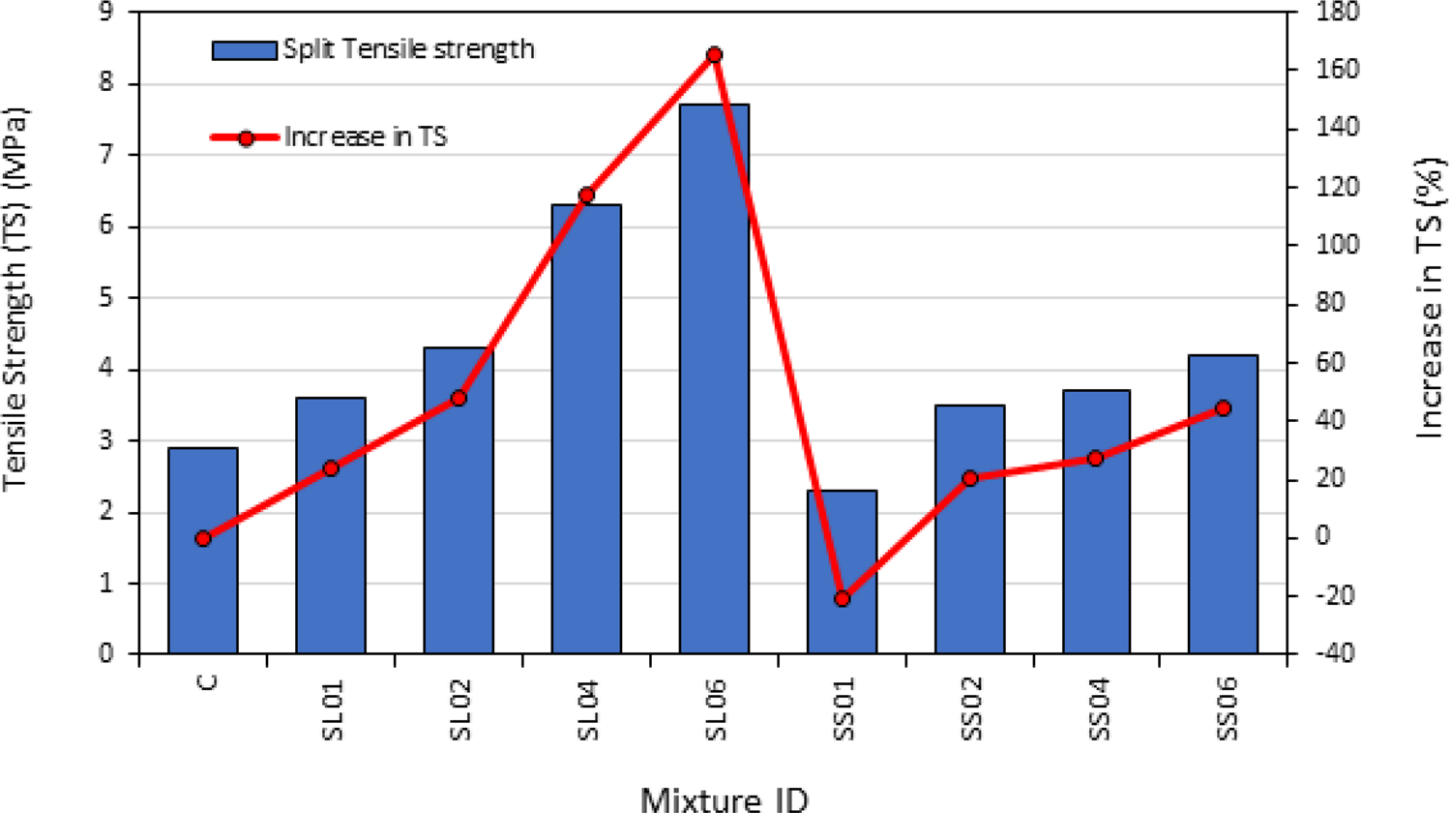
Tensile strength results of PASFRC.

The splitting tensile strength test results after 28-day for PASFRC mixtures are presented in Table 6 and Fig. 11. The split tensile strength varied between 3.8 MPa and 6 MPa dependent on fiber dosage. As predicted, the steel fibers showed significant effect on the tensile strength of the PASFRC specimens. PASFRC mixtures that contained various proportions of steel fibers, displayed superior tensile strength values compared to the plain PAC mixture. As a result, the addition of long steel fibers (i.e., SL01, SL02, SL04, SL06) at doses of 1%, 2%, 4% and 6% in the mixtures led to a considerable increase in tensile capacity of 24.1%, 48.3%, 117.2% and 165.5% respectively, as compared to the control mixture C. The result was contrasted with a similar finding presented by Nehdi et al.^31^. Both studies observed enhancement in tensile capacity which can be ascribed to the interfacial bond between the matrix and fibers. This enhanced bond facilitates the transfer of load across cracks, resulting in an overall improvement in the ability to carry tensile loads. The increase in fiber content further enhances this load transfer mechanism, resulting in an overall improvement in tensile load carrying capacity.

PASFRC mixtures with a high steel fiber dosage (more than 2%), the fibers tend to exhibit a random distribution inside the preplaced aggregate matrix. This random distribution leads to the occurrence of many fibers intersecting any potential failure plane. Therefore, an increased dosage of fiber led to a corresponding increase in tensile strength^73^. The SL04 and SS04 combinations demonstrated a respective increase of 61.9%, and 83.7% in tensile strength in comparison to the control mixture. Furthermore, the tensile strength of PASFRC mixtures containing a long hooked-end fiber dosage of 6% exhibited more than 2.5 times the strength of the control mixture (165.5% increase). This phenomenon described can be ascribed to the effect of steel fibers, which effectively intersect, block, and limit the spread of cracks^74^. Nevertheless, the incorporation of short hooked end fibers in PAC mixtures led to a small increase in tensile strength compared to PAC’s control specimen. As seen on Fig. 11, the tensile strength of SS02, SS04, and SS06 mixtures exhibited an increase of around 20.7%, 27.6%, and 44.8% respectively, when compared to the control mixture. The confirmation of these finding was carried out using ANOVA, which verified that the variation in fiber dosage had a statistically significant impact on the mean of the tensile strength findings. It revealed a significantly greater F value of 86.69 compared to the critical F value of 7.71 $({F}_{\mathrm{0.05,1},4})$ and a P-value of 0.0007 was obtained, which indicates the result was significant compared to the significance level of 0.05. This also indicates the fiber dose is a significant factor in the model as shown in Table 7.

The study's results indicated that the usage of long hooked end steel fibers, resulted in higher compressive strength when compared to the shorter hooked end steel fibers. In the case of SL04, SL06 PASFRC mixtures, which were reinforced with a steel fiber dosage of 4% and 6%, displayed a notable difference in tensile strength of 70.3% and 83.3% compared to SS04 and SS06 mixtures, respectively. ANOVA (Table 7) provided evidence supporting the results that the variability in fiber length had a statistically significant impact on the tensile strength results. The obtained F value of 80.77 for the tensile strength of PASFRC was found to be significantly higher than the crucial F value of 7.71 $({F}_{\mathrm{0.05,1},4})$. Also, P-value of 0.0008 was found to be significantly lower than the significant level 0.05 which indicate that the fiber length is a significant model term. Based on ANOVA’s analysis the absolute fraction of variance (R^2^) was 0.9778. Therefore, an empirical equation for predicting the tensile strength of PASFRC is proposed based on certain dosages and steel fiber lengths.

6 $${f}_{t}= 1.86271+0.014576\mathrm{l }+0.020746{f}_{d}$$

where ${f}_{t}$= tensile strength of PASFRC (MPa); ${\text{l}}$= steel fiber length (i.e., 35 or 60 mm); and ${f}_{d}$= Steel fiber dosage (i.e., 1,2,4, or 6%).

The observed increase in post-cracking strength in PASFRC mixtures consisting of long fibers can be attributed to the presence substantial internal voids within the PAC aggregates, which arise from the bleeding of the grout mixture^75^. Hence, the presence of these voids can have a negative effect on the binding between the fiber and matrix, particularly when dealing with short crimped steel fibers, resulting in a decrease in tensile strength. Long hooked end steel fibers exhibited superior macrocrack bridging capabilities and effective bonded length compared to short fibers, leading to an increase in tensile strength^76^.

### Flexural strength

**Figure 12:**
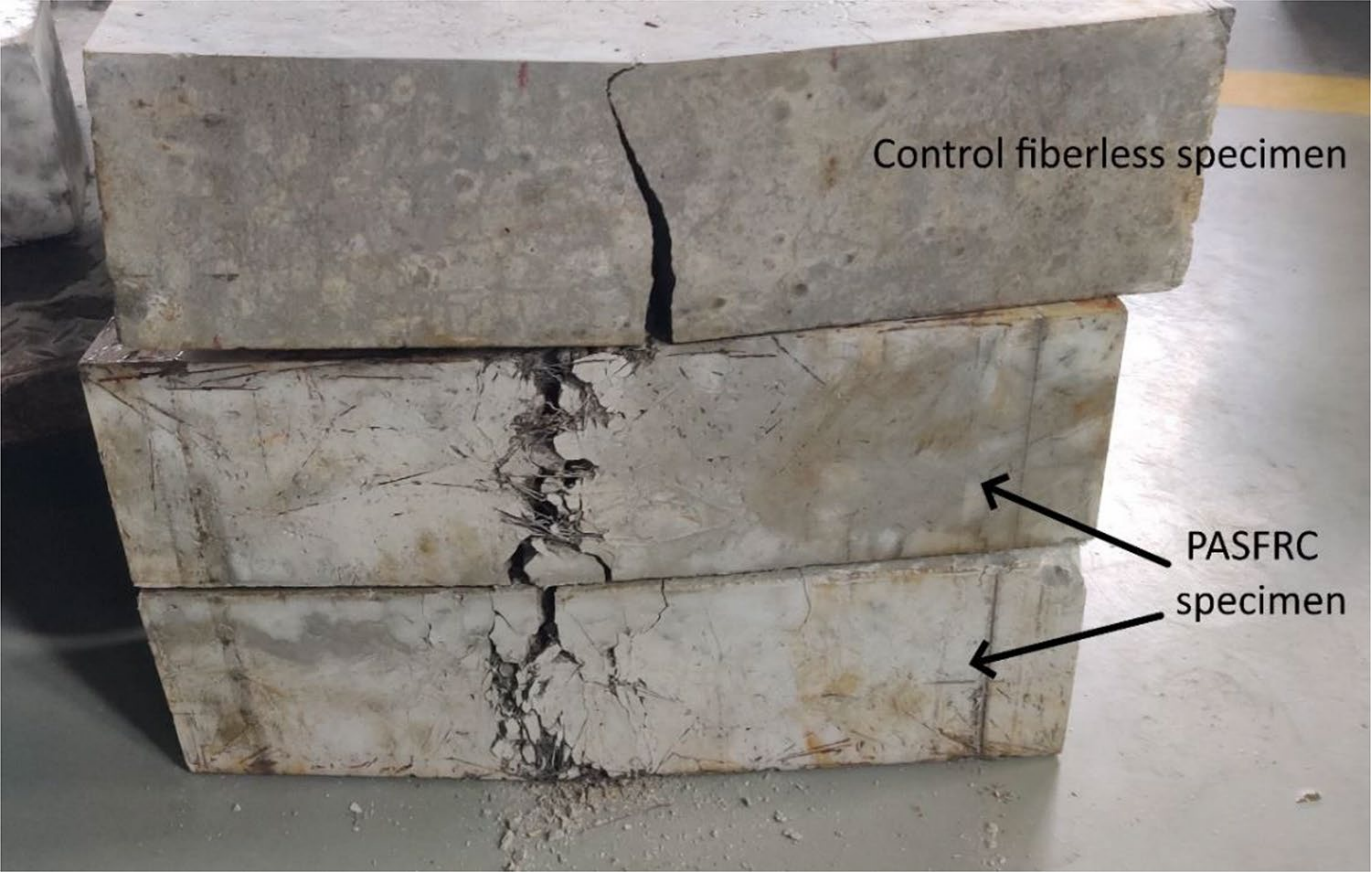
Flexural failure mode of both fiberless and steel reinforced PASFRC specimens.

Table 6 presents the outcomes of the flexural strength test conducted on PASFRC specimens, which were produced using various dosages of steel fibers comprising two different lengths. Similar to traditional concrete, an addition of steel fibers affected the failure behavior of the investigated mixtures, shifting its failure mode from a brittle to a ductile failure mode. The failure mode difference between ductile steel fiber reinforced specimens and brittle fiberless specimen is shown in Fig. 12. The inclusion of steel fibers enhanced the adhesion between the matrix and fibers in the PASFRC. The fibers acted as bridges across the microcracks, effectively restraining crack propagation. This bridging effect can be clearly seen from the crack widths observed in the failure modes displayed in Fig. 12. Fiberless PAC specimens revealed sudden failure at peak loads, accompanied by the presence of extensive cracks wide crack widths. In PASFRC specimens, the crack width steadily expanded till reaching the ultimate load, with both fractured prisms remaining. Previous research studies have shown a similar type of failure^54,61,77^.

**Figure 13:**
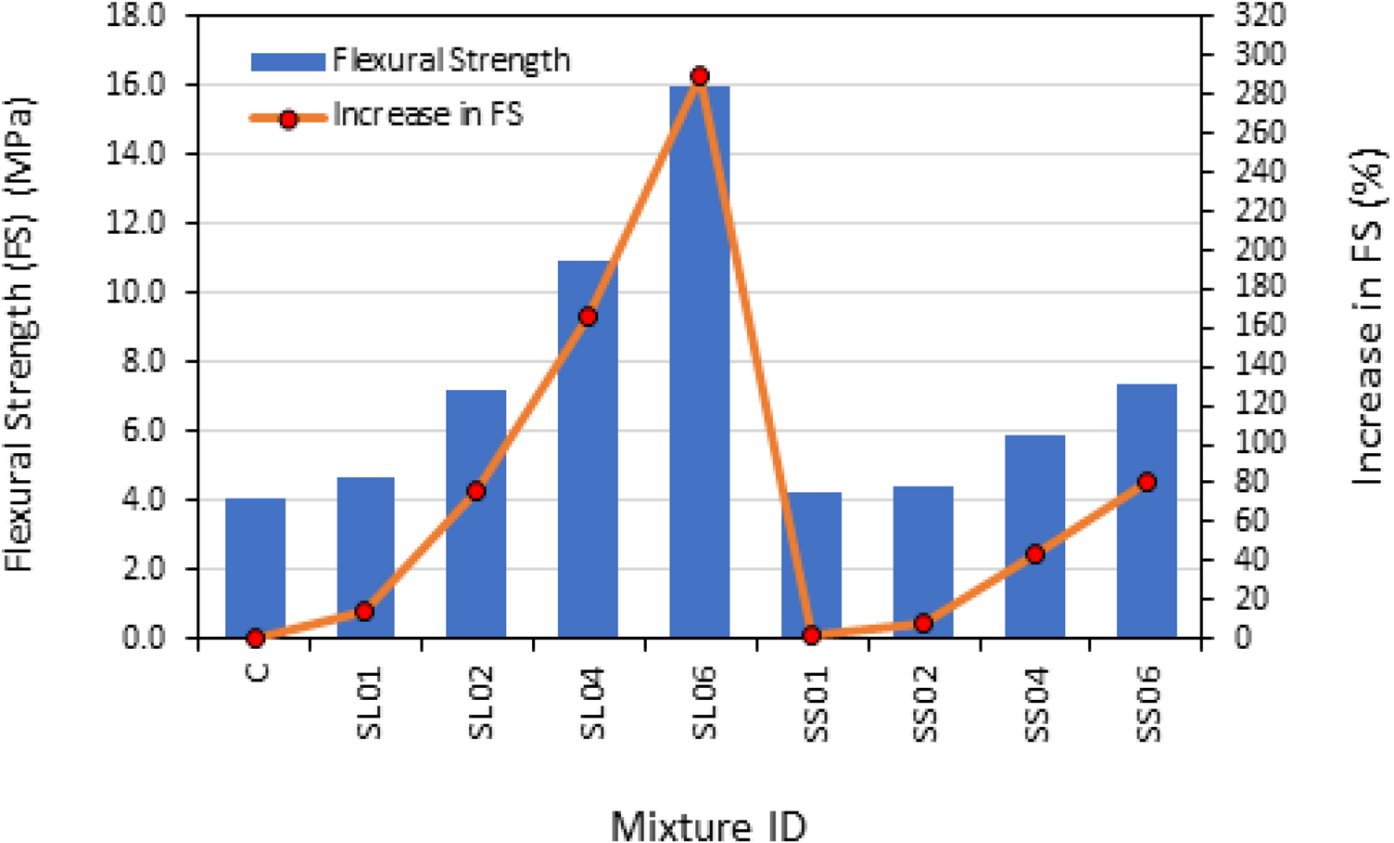
PASFRC flexural strength results.

As predicted, the flexural strength of PASFRC demonstrated a notable enhancement upon the incorporation of long hooked end steel fibers. As a representative example, Fig. 13 shows the flexural strength of the SL02, SL04 and SL06 mixtures exhibited an increase of 75.6%, 165.9% and 290.2% compared to the fiberless control mixture C. Additionally, the validity of these findings was confirmed through a statistical analysis, as demonstrated by the outcomes presented in Table 7. ANOVA conducted on the flexural strength data resulted in a F value of 540.5, which is greater than the corresponding F value of 7.71 $({F}_{\mathrm{0.05,1},4})$. The findings indicate that the change in fiber dosage had a significant effect on the PAC flexural strength results.

whereas it was observed that the addition of short steel fibers at low doses (1% and 2%) resulted in a minor increase in flexural strength. Particularly, SS01 and SS02 showed a respectively increase of merely 2.4% and 7.3% in flexural strength compared to the control mixture. mixtures S01 (containing 1% short steel fibers) and SS02 (containing 2% short steel fibers) displayed lower flexural strength by 11.9% and 63.6% compared to specimens obtained from mixtures SL01 and SL02, which incorporated equivalent amounts from the short fiber mixtures, respectively.

**Figure 14:**
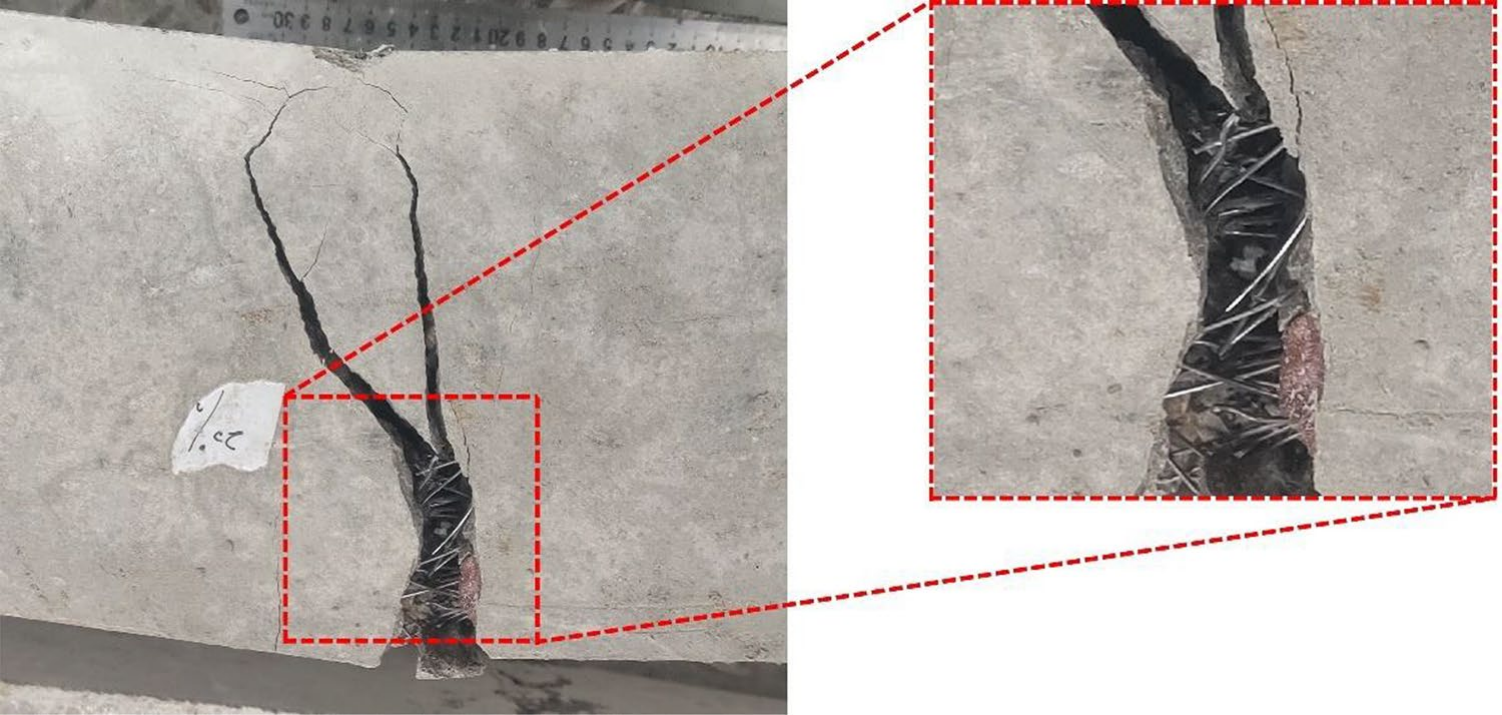
Crack pattern of PASFRC sample shows that long steel threads are bridging cracks.

In general, the flexural strength of PASFRC with long steel fibers measuring 60 mm was found to be higher compared to PASFRC including shorter fibers measuring 35 mm. As shown in Table 6, the flexural strength of the SL04 and SL06 mixtures exhibited an increase of 84.7% and 116.2% respectively, in comparison to the SS04 and SS06 mixtures. It concludes that the residual strength of long hooked end steel fibers is higher compared to that of short hooked end steel fibers. Additionally, the prevention of microcrack formation and propagation can be attributed to the efficient bonded length and bridging action exhibited by the long steel fibers. Also, steel fibers with longer lengths have an increased average embedment length, hence leading to enhanced resistance against pull-out forces^31^. Figure 14 illustrates the fracture bridging mechanism seen in a PASFRC specimen consisting of long steel fibers. According to the research conducted using ANOVA, the absolute fraction of variance (R^2^) was found to be 0.8428. Hence, a proposed empirical equation is presented for the estimation of the flexural strength of PASFRC. The equation below is derived from the used dosages and lengths of steel fibers, namely 1%, 2%, 4%, or 6% dosages and fiber lengths of 35mm or 60mm:

7 $${f}_{r}= -5.11394 + 0.169\mathrm{ l }+1.43814{f}_{d}$$

where ${f}_{r}$= Modulus of rupture (flexural strength) of PASFRC (MPa); ${\text{l}}$= steel fiber length (i.e., 35 or 60 mm); and ${f}_{d}$= Steel fiber dosage (i.e., 1, 2, 4, or 6%).

According to the ANOVA results in Table 7, the change in fiber length had a significant effect on the mean of flexural strength, as indicated by the computed F value of 370.43, which was greater than the equivalent critical F value (F0.05, 1, 6) of 7.71. The P-value was less than 0.0001 which indicate that the result was significant compared to the significance level of 0.05. This also indicates the fiber dose is a significant factor in the model.

Table 8 presents a comparative analysis of the increases observed in the compressive, tensile, and flexural strengths of different concrete types from previous research studies with relation to this current PASFRC study. The results indicate that the incorporation of long steel fibers measuring 60 mm in length in PASFRC led to a notable improvement in flexural strength when compared to both ultra-high performance fiber reinforced concrete (UHPFRC)^78^ and Two-Stage Fiber-Reinforced Concrete (TSFRC)^40^. The PASFRC mixtures containing 6% long steel fibers measuring 60 mm in length displayed a significant increase in compressive, tensile, and flexural strengths. Specifically, these mixtures exhibited approximately 262% higher compressive strength, 36% higher tensile strength, and 9% higher flexural strength compared to two-stage steel fiber-reinforced concrete (PASFRC) mixtures incorporating 6% long steel fibers of the same length^31^. This underscores the notable advantages that may be conveyed through the utilization of Portland limestone cement (PLC) in the preplaced aggregate concrete PAC technique.

**Table 8:** Increase in compressive, tensile and flexural strengths of different types of concrete with steel fibers compared with their control plain concrete counterpart.

Concrete type	Steel fiber length (mm)	Steel fiber dosage (Vol. %)	Increase in compressive strength (%)	Increase in Tensile strength (%)	Increase in flexural strength (%)
HSFRC^a^^79^	19	1	11	34	8
		2	19	56	18
		3	26	88	28
		4	8	91	23
TSSFRC^b^^31^	33	1	14	30	22
		2	18	59	41
		4	30	81	78
		6	49	108	149
	60	1	15	35	8
		2	22	68	30
		4	31	97	157
		6	52	122	243
UHPFRC^c^^78^	16	1	5	48	37
		3	9	101	112
		6	13	260	223
TSFRC^40^	15	1.5	18	17.1	9.5
		3	28	34.2	14.9
		5	41	43.9	28.9
	30	1.5	28	29.5	16.4
		3	38	61.9	22.6
		5	54	83.7	41.7
TSFRC^54^	20–45*	0.5	− 5.1	44.7	–
		1	− 6	50.8	–
		1.5	− 6.4	60.5	–
PASFRC	35	1	19	− 21	− 5
		2	68	21	0
		4	126	28	34
		6	154	45	68
	60	1	75	24	7
		2	109	48	64
		4	161	117	148
		6	188		

^a^HSFRC refers to high-strength fiber-reinforced concrete.

^b^TSSFRC refers to two-stage steel fiber-reinforced concrete.

^c^UHPFRC refers to ultra high performance fiber-reinforced concrete.

*Steel wires ranging between 20 and 45 mm.

## Conclusions

The present study pioneered the concept of incorporating Portland limestone cement in preplaced aggregate steel fiber reinforced concrete (PASFRC) mixtures. Based on the findings obtained, the following conclusions can be drawn:

1. The research introduces a novel approach to develop a sustainable preplaced aggregate fiber reinforced concrete by incorporating PLC, thereby reducing CO2 emissions through the reduction of energy consumption during cement manufacturing. In addition, due to PASFRC sustainable low energy mixing and strategic placement this resulted in a more sustainable type of concrete.
2. On the contrary to SFRC, the production of PASFRC allows for the addition of steel fibers up to 6%, resulting in outstanding mechanical properties. It was noticed that the increase of steel fibers doses had a significant improvement on the mechanical properties of PASFRC. Also, the addition of long end-hooked steel fibers resulted in a significant improvement compared to the addition of short end-hooked steel fibers.
3. The PASFRC mixtures with a long steel fiber dosage of 6% exhibited the highest values of compressive, tensile, and flexural strengths, measuring 49.8 MPa, 7.7 MPa, and 10.9 MPa, respectively. Compared to fiberless PAC these strengths had a significant difference of 188%, 166%, and 290%, respectively.
4. ANOVA proved that adding steel fibers to the PAC mixtures at different doses and two different lengths significantly improved PAC’s mechanical properties. The test specimens with the highest compressive, tensile, and flexural strengths were SL06 (6% long steel fibers). Based on the used steel fibers new empirical equations were proposed to predict the compressive, tensile and flexural strengths of PASFRC.

PASFRC, that uses PLC, is a novel concept for PAC offering a wide range of potential applications. There are still several significant aspects and properties that need to be investigated. Future research should explore different sand to binder ratios, water to binder ratios, and the incorporation of polypyrene fibers. Future research could capitalise on analysing the impact resistance, durability, and abrasion resistance of PAFRC by experimental and modelling analysis.

## Data Availability

The datasets used and/or analysed during the current study available from the corresponding author on reasonable request.
